# Spatial-temporal evolution and influencing factors of sudden environmental accidents in China from 2008 to 2022

**DOI:** 10.1371/journal.pone.0339526

**Published:** 2026-01-05

**Authors:** Jun Yan, Shi Yan, Shihan He, Xinying Wang, Xuemei Yang, Xu Li

**Affiliations:** 1 School of Geographic Sciences, Xinyang Normal University, Xinyang, China; 2 Henan Key Laboratory for Synergistic Prevention of Water and Soil Environmental Pollution, Xinyang Normal University, Xinyang, China; Chang'an University, CHINA

## Abstract

The spatiotemporal evolution characteristics and influencing factors of sudden environmental accidents are analyzed by employing exploratory spatial analysis and Pearson correlation analysis, based on the statistical data of sudden environmental accidents in 31 provinces, municipalities, and autonomous regions in China from 2008 to 2022. The results provide valuable insights for the prevention and treatment of sudden environmental pollution emergencies. The results showed that: (1) the total of sudden environmental accidents exhibited an inverted V shaped structure with 2013 as the turning point, showing an overall decreasing trend in China. (2) The seasons when the sudden environmental accidents occurred from the highest to lowest proportions were autumn (29.01%), summer (26.29%), spring (24.80%), and winter (20.19%). The months with the highest frequency were May and July, while October and December had the lowest. The dates with the most occurrences were the 5th, 7th, 4th, 2nd, 11th, and 9th, while the 31st, 30th, 29th, and 27th were the lowest. Regarding weekdays, Monday (16.56%), Wednesday (15.94%), and Thursday (14.38%) were the highest proportions, while Sunday (12.50%) was the lowest. (3) Spatial distribution revealed an overall imbalance, with the eastern coastal comprehensive economic zone being the highest frequency of environmental accidents, followed by the middle reaches of the Yellow River comprehensive economic zone, and the northeast comprehensive economic zone having the lowest. Provinces with the highest number of sudden environmental accidents were mainly in Shanghai (1129), Shaanxi Province (472), and Jiangsu Province (419). Based on the cold and hot spot analysis, H-H areas were mainly located in the southeast coastal regions, L-L areas were concentrated in the western and northeastern regions, and L-H areas were distributed in the central regions. (4) Pearson correlation analysis indicated that investment in the treatment of industrial pollution as percentage of GDP and secondary industry output value as Percentage of GDP were the main driving factors for sudden environmental accidents in China. Per capita GDP, pollutant emissions, and the total of letters and phone calls regarding environmental pollution had inhibitory effects on the occurrence of sudden environmental accidents, while single factors had relatively minor impacts. To effectively prevent and control sudden environmental accidents, it is necessary to improve the risk management system for sudden environmental accidents and strengthen monitoring and management of accident-prone industries, dates, and regions.

## 1. Introduction

Sudden environmental accidents refer to incidents triggered by factors such as pollution discharge, natural disasters, or production safety accidents. These events cause toxic and harmful substances—including pollutants or radioactive materials—to enter environmental media (e.g., the atmosphere, water bodies, soil, etc.), leading to abrupt degradation of environmental quality. Such accidents may endanger public health and property safety, inflict ecological environmental damage, or generate significant social impacts, thus necessitating the implementation of emergency response measures [1]. Major pollution accidents pose a serious threat to environmental quality and public health, which attain wide media coverage and even provoke public panic [2]. In recent decades, environmental risks have become prominent in China with the development of the economic and social commission; all kinds of sudden environmental pollution accidents have occurred frequently. It is obvious that the spatial distribution characteristics of sudden environmental incident are regional clustering, and the situation of environmental emergency response is still grim [3]. Due to the highly random, strong destructive and uncertain nature of sudden environmental events, especially major ones, they pose serious threats to public health and property safety and significantly impact China’s ecological civilization construction. It is a challenging and inevitable topic for academic research to study spatial-temporal evolution and influencing factors of sudden environmental accidents and effectively prevent and deal with the occurrence of ones. The appropriate emergency response can minimize the harmful effects of accidents and protect people’s lives and property [4].

Some scholars at home and abroad have carried out extensive research on sudden environmental accidents while paying attention to the frequent occurrence of sudden environmental accidents in recent years. Developed nations such as the United States and Japan initiated research on emergency preparedness for sudden environmental accidents in the early 20th century [5]. These countries have established a comprehensive environmental management framework for emergency response, encompassing risk assessment and policy management systems, multi-level linkage emergency response mechanisms, and information exchange platforms—all of which underpin emergency decision-making for sudden environmental incidents [6]. Following the promulgation of the Contingency Plan for Environmental Emergencies by China’s State Council in 2006, a risk emergency management system for sudden environmental accidents has been preliminarily established [7]. Most domestic scholars have studied sudden environmental accidents from three aspects: Firstly, Scholars have conducted extensive research on response mechanisms for sudden environmental incidents, encompassing emergency monitoring [8], risk assessment [9], pollution source tracing [10], early warning systems [11], damage assessment [12], emergency management [13], impact analysis [14], and problem prevention strategies [15]. Key findings indicate that sudden environmental accidents exhibit high-risk characteristics marked by complex scenarios, diverse incident types, intricate causal factors, high public visibility, and frequent occurrences [16]. Successful response experiences highlight several critical components: robust grass-roots environmental emergency preparedness, an efficient and streamlined emergency command system, a sound early warning and prevention mechanism, strong scientific-technological support with adequate material stockpiling/transportation guarantees, and a transparent information disclosure framework.Secondly, the spatial-temporal characteristics of sudden environmental accidents are studied. Sudden environmental accidents in China generally show a downward trend in time, and the high-frequency risk areas in space gradually decrease, and the high-frequency areas are concentrated in the pan-triangle area. Yu Guanghui et al. employed spatial autocorrelation and spatiotemporal geographical weighted regression (GTWR) models to analyze the spatial dependence of sudden environmental accidents and the spatiotemporal heterogeneity of influencing factors [17]. Using GIS technology and non-parametric correlation analysis, Li Jing et al. investigated the dynamic change trends and spatial distribution patterns of environmental pollution and damage accidents in China [18]. Ding Lei et al. applied ESDA (Exploratory Spatial Data Analysis) techniques and MATLAB-based spatial panel econometric models to explore the spatiotemporal evolution characteristics and driving factors of environmental pollution incidents across Chinese regions during 1995–2012 [19]. Li Xu et al. applied Origin software from six aspects to carry out statistics and analysis on 3203 sudden environmental accidents in China from 2011 to 2017 [20]. The Kernel Density and spatial mean center of sudden water pollution incidents transfer curve were used to explore the characteristics [21].Thirdly, the influencing factors of sudden environmental accidents are studied. The main influencing factors are regional population size, economic development level, industrial structure, pollution control level and legal environment, etc. The influencing factors show obvious spatial and temporal heterogeneity in different periods and different provinces [22]. The disaster types are dominated by water pollution and air pollution accidents [23].

In summary, previous studies were mainly conducted from three aspects, such as event response, characteristic evolution, and influencing factors of sudden environmental accidents. However, further studies are still needed on the driving mechanism of regional sudden environmental accidents and the refinement of spatial and temporal distribution. Therefore, the research aims to explore further the spatial-temporal evolution characteristics and driving mechanism of sudden environmental accidents in China based on exploratory geographic analysis and Pearson correlation analysis from 2008 to 2022, such as the research scales of year, quarter, date and regional distribution, in order to expand a new perspective for the study of human-land system changes and provide decision-making reference for China’s ecological civilization construction in the new development stage.

## 2. Materials and methods

### 2.1. Data source

The data of sudden environmental accidents and other related economic indicators were mainly obtained from China Statistical Yearbook [24], China Environmental Statistical Yearbook [25] and the statistical yearbooks of each province from 2009 to 2023 in China. Hong Kong, Macao, and Taiwan are not included in the sample set due to missing data. The specific occurrence time data of sudden environmental accidents are derived from statistics of environmental events of the Journal of Safety and Environment from 2008 to 2022 [26]. This paper takes the eight comprehensive economic zones delineated in the report Strategies and Policies for Coordinated Regional Development by the Development Research Center of the State Council as the research object. The range of eight economic zones in China are Northeast economic zone (NEEZ) including Liaoning, Jilin, Heilongjiang; Northern coastal economic zone (NEZ) including Beijing, Tianjin, Hebei, Shandong; Eastern coastal economic zone (ECEZ) including Shanghai, Zhejiang, Jiangsu; Southern coastal economic zone (SCEZ) including Fujian, Guangdong, Hainan; Mid-Yellow river economic zone (MYeREZ) including Shanxi, Inner Mongolia, Henan, Shaanxi; Mid-Yangtze river economic zone (MYtREZ) including Hubei, Hunan, Jiangxi, Anhui; Southwest economic zone (SWEZ) including Yunnan, Guizhou, Sichuan, Chongqing, Guangxi; and Northwest economic zone(NWEZ) including Gansu, Qinghai, Ningxia, Xinjiang. The data source is shown in Table 1.

**Table 1:** Data source.

Data	Data Sources	Time Series
Number of sudden environmental accidents	CHINA STATISTICALYEARBOOK、 CHINA STATISTICAL YEARBOOK ON ENVIRONMENT	2008 - 2022
Map	GS(2020)4619	2020
Per capita GDP	CHINA STATISTICALYEARBOOK、National Bureau of Statistics of China	2008- 2022
Investment in the treatment of industrial pollution as percentage of GDP	CHINA STATISTICALYEARBOOK、National Bureau of Statistics of China	2008 - 2022
Secondary industry output value as Percentage of GDP	CHINA STATISTICALYEARBOOK、National Bureau of Statistics of China	2008 - 2022
Pollutant emissions	CHINA STATISTICALYEARBOOK、National Bureau of Statistics of China	2008 - 2022
The total of letters and phone calls regarding environmental pollution	CHINA STATISTICALYEARBOOK、National Bureau of Statistics of China	2008 - 2022

### 2.2. Research method

#### 2.2.1. Spatial statistical analysis:

Spatial autocorrelation refers to the statistical dependency of variables across different spatial positions, typically categorized as positive correlation (aggregation), negative correlation (dispersion), or non-correlation (randomness), encompassing both global and local spatial autocorrelation frameworks analyzed via distinct indices. Global spatial autocorrelation characterizes the overall spatial distribution pattern of an attribute value across a study region [27], with key metrics including Global Moran’s I, Global Geary’s C, and Global Getis-Ord G—all evaluating spatial autocorrelation by measuring similarity of observed values between neighboring units. This study employed the Global Moran’s I coefficient for autocorrelation analysis, with its calculation formula as follows:


$$\begin{array}{c} I=\frac{n\sum_{i=\text{1}}^{n}\sum_{j=\text{1}}^{n}w_{ij}(x_{i}-\overset{―}{x})(x_{j}-\overset{―}{x})}{(\sum_{i-\text{1}}^{n}\sum_{j=\text{1}}^{n}w_{ij})\sum_{i=\text{1}}^{n}{\left(x_{i}-\overset{―}{x}\right)}^{\text{2}}},\;\;\overset{―}{x}=\frac{\text{1}}{n}\sum {\mathrm{\backslash nolimits}}_{i=\text{1}}^{n}x_{i} \end{array}$$ (1)

Where, *I* is the global Moran index; *n* is the total number of the studied regions, *W*_*ij*_ stands for the space weight matrix, it reflects the spatial relationship between regions *i* and *j*, and it is defined as: if it is adjacent, *W*_*ij*_ = 1, otherwise, *W*_*ij*_ = 0; *x*_*i*_ and *x*_*j*_ are the observed values of studied region *i* and *j*, respectively, andx denotes the average values in the study area. The value range of the global Moran index I is [−1, 1]. For Moran index, the standardized statistic *Z* can be used to test whether there is a spatial autocorrelation between n regions. The formula for *Z* is as follows:


$$Z=\frac{I-E(I)}{\sqrt{VAR(I)}}=\frac{\sum_{j\neq i}^{n}w_{ij}(d)(x_{j}-{\bar{x}}_{i})}{S_{i}\sqrt{w_{i}(n-\text{1}-w_{i})/(n-\text{2})}},j\neq i$$ (2)

E(I) and VAR(I) represent its theoretical expectation and variance, with the mathematical expectation given by E(I) = −1/(n-1). A positive and significant Z-value indicates positive spatial autocorrelation, where similar observations (either high or low) tend to cluster; a negative and significant Z-value signifies negative spatial autocorrelation, with similar observations dispersing; and a Z-value of zero implies observed values are distributed independently and randomly.

#### 2.2.2. Pearson correlation analysis:

Pearson’s correlation coefficient (r) is a measure of the linear association of two variables. The Pearson correlation coefficient is calculated as follows:


$$\text{r}=\frac{\sum_{i=\text{1}}^{n}\left(x_{i}-\overset{―}{x})(y_{i}-\overset{―}{y}\right)}{\sqrt{\sum_{i=\text{1}}^{n}{\left(x_{i}-\overset{―}{x}\right)}^{\text{2}}\times \sum_{i=\text{1}}^{n}{\left(y_{i}-\overset{―}{y}\right)}^{\text{2}}}}$$ (3)

Where, X_i_ represents independent variables—including per capita GDP, the ratio of industrial pollution treatment volume to GDP, the secondary industry output value-to-GDP ratio, pollutant emissions, and the total number of environmental pollution-related letters and phone calls—and other factors influencing sudden environmental accidents, and Y_i_ denotes the dependent variable (i.e., the number of sudden environmental accidents). The Pearson correlation coefficient is standardized by dividing by the product of the variables’ standard deviations to measure the proportion of covariance relative to individual variability, fundamentally assessing correlation through covariance: positive when variables deviate from their means in the same direction, negative in opposite directions. Ranging between −1 and +1, it is interpreted as: + 1 for perfect positive linear relationship (more of one variable corresponds to more of the other), −1 for perfect negative linear relationship (more of one corresponds to less of the other), and 0 for no linear relationship (independent random distribution).

## 3. Results and discussion

### 3.1. Temporal evolution characteristics of sudden environmental accidents

#### 3.1.1. Annual changes of sudden environmental accidents.

The classification criteria for sudden environmental incidents in China are mainly based on the National Emergency Plan for Sudden Environmental Incidents (revised in 2015), which categorizes incidents into four levels: Especially Serious, Major, Relatively Major, and General. The specific classification standards comprehensively consider core factors including casualties, evacuation and relocation, economic losses, radiation pollution,and impact (Table 2).

**Table 2:** The classification criteria for sudden environmental incidents in China.

Sudden environmental accidents	Casualties	Evacuation and relocation	Economic losses	Impact	Radiation pollution	Other impacts
Extraordinarily serious environmental emergencies events (Level Ⅰ)	Directly causing 30 or more deaths, or 100 or more cases of poisoning/serious injury due to environmental pollution.	Requiring the evacuation and relocation of 50,000 or more people.	Direct economic losses exceeding 100 million yuan.	Resulting in the loss of regional ecological functions or the extinction of national key protected species.	Loss, theft, or loss of control of Class Ⅰ or Ⅱ radioactive sources leading to large-scale and serious radiation pollution; leakage of radioactive substances causing 3 or more acute deaths or large-scale radiation pollution.	Causing significant transnational environmental impacts.
Serious environmental emergencies events (Level Ⅱ)	Directly causing 10 to 30 deaths, or 50 to 100 cases of poisoning/serious injury.	Requiring the evacuation and relocation of 10,000 to 50,000 people.	Direct economic losses ranging from 20 million yuan to 100 million yuan.	Resulting in partial loss of regional ecological functions or mass death of populations of national key protected wild animals and plants.	Loss or theft of Class Ⅰ or Ⅱ radioactive sources; leakage of radioactive substances causing fewer than 3 acute deaths or 10 or more cases of acute severe radiation sickness.	Causing environmental impacts across provincial administrative regions.
Comparatively serious environmental emergencies events (Level Ⅲ)	Directly causing 3 to 10 deaths, or 10 to 50 cases of poisoning/serious injury.	Requiring the evacuation and relocation of 5,000 to 10,000 people.	Direct economic losses ranging from 5 million yuan to 20 million yuan.	Causing damage to national key protected animal and plant species.	Loss or theft of Class Ⅲ radioactive sources; leakage of radioactive substances causing fewer than 10 cases of acute severe radiation sickness.	Causing environmental impacts across prefecture-level administrative regions.
Ordinary environmental emergencies events (Level Ⅳ)	Directly causing fewer than 3 deaths, or fewer than 10 cases of poisoning/serious injury.	Requiring the evacuation and relocation of fewer than 5,000 people.	Direct economic losses of less than 5 million yuan.	Triggering disputes across county-level administrative regions or general mass incidents.	Loss or theft of Class Ⅳ or Ⅴ radioactive sources; leakage of radioactive substances causing local pollution within factory areas.	Causing certain environmental impacts but not meeting the standards for relatively major incidents.

The total of 4,736 cases of sudden environmental accidents was reported in China from 2008 to 2022 according to Ministry of Ecology and Environment of China. The average annual cases of sudden environmental accidents reported were 316. The maximum values occurred in 2013 with 712 accidents, accounting for 15.03% of the total. The lowest frequency was 113 cases in 2022, accounting for 2.39% of the total. The State Council is-sued a new contingency plan for environmental emergencies on December 29, 2014.Sudden environmental accidents are divided into four levels according to the severity and urgency of emergencies: extraordinarily serious environmental emergencies events (Level Ⅰ); serious environmental emergencies events (Level Ⅱ); comparatively serious environmental emergencies events (Level Ⅲ) and ordinary environmental emergencies events (Level Ⅳ). There were 4423 cases (93.39%) of sudden environmental accidents, which were mainly ordinary environmental emergencies events (Ⅳ). Comparatively serious environmental emergencies events(Ⅲ) were 198 (4.27%) (Table 3).

**Table 3:** The total of sudden environmental accidents from 2008 to 2022.

Year	Level I	Level II	Level III	Level IV	Not Applicable (N/A)	Total
2008	0	12	31	92	0	135
2009	2	2	41	126	0	171
2010	0	5	41	109	1	156
2011	0	6	10	465	62	542
2012	0	5	5	532	0	542
2013	0	3	12	697	0	712
2014	0	3	16	452	0	471
2015	0	3	5	326	0	334
2016	0	3	5	296	0	304
2017	0	1	6	295	0	302
2018	0	2	6	278	0	286
2019	0	0	3	258	0	261
2020	0	2	8	198	0	208
2021	0	2	9	188	0	199
2022	0	2	0	111	0	113
Total	2	51	198	4423	62	4736

Annotation: Extraordinarily Serious Environmental Emergencies events (Level Ⅰ); Serious Environmental Emergencies events (Level Ⅱ); Comparatively Serious Environmental Emergencies events (Level Ⅲ); Ordinary Environmental Emergencies events (Level Ⅳ). Not Applicable (N/A).

The total number of sudden environmental accidents shows an overall downward trend in China from 2008 to 2022. The quantity of sudden environmental accidents in China exhibited an inverted V shaped structure with 2013 as the turning point. The number of sudden environmental accidents decreased significantly after 2014, and the rate of increase and decrease is first fast and then slow.

#### 3.1.2 Seasonal variation of sudden environmental accidents.

The seasonal distribution mainly concentrated in summer and autumn, and autumn accounted for the largest proportion. The seasonal variations of sudden environmental accidents from high to low was autumn (29.01%), summer (26.29%), spring (24.80%) and winter (20.19%) in China from 2008 to 2022.

#### 3.1.3 Monthly change rules of sudden environmental accidents.

**Fig 1:**
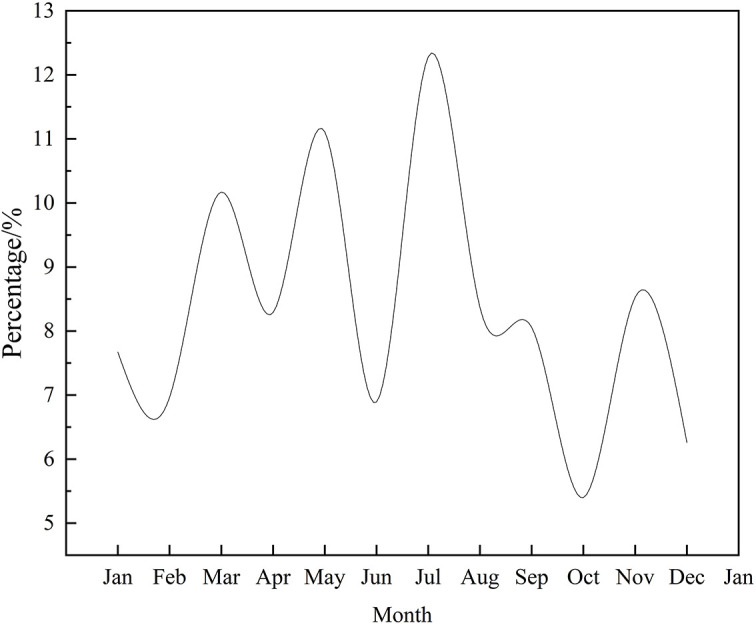
Monthly changes of sudden environmental accidents of China from 2008 to 2022.

From 2008 to 2022, the monthly distribution of sudden environmental accidents in China showed that the highest proportion occurred in July (12.28%), and the lowest in December (6.26%). Other months with relatively high proportions included May (11.11%) and March (10.17%). Months with lower proportions were October (5.40%), December, June (6.89%), and February (6.96%). The proportions for the remaining months fluctuated around 8% (Fig 1).

#### 3.1.4 Daily variation rules of sudden environmental accidents.

Based on daily statistical data from 2008 to 2022, the date with the highest frequency of sudden environmental accidents was the 5th of the month, which recorded 58 occurrences. This was followed by the 7th (57 times), 4th (54 times), 2nd (52 times), 11th (51 times), and 9th (50 times), all of which exceeded 50 occurrences. At the other end of the spectrum, the 31st had the lowest frequency, with only 14 cases. Other dates with fewer than 30 occurrences included the 30th (24 times), 29th (24 times), and 27th (30 times).In terms of weekly patterns, the top three days for sudden environmental accidents were Monday (16.56%), Wednesday (15.94%), and Thursday (14.38%), while Sunday had the lowest proportion at just 12.50%. Overall, the temporal distribution of such accidents in China shows a clear concentration at the start of each month, with a significant decrease in occurrences toward the end of the month.

### 3.2 Spatial distribution characteristics of sudden environmental accidents

#### 3.2.1 Geographical distribution characteristics.

China has a vast territory in contrast to most other countries, and the level of economic development varies greatly between regions, especially obvious imbalances also exist within some sectors and provinces. For a long time, China’s regional economic development has mainly focused on the four major sectors: East China, Central China, West China and Northeast China. However, China’s regional economic development has obvious differentiation trend, and there is also obvious differentiation within each sector. Therefore, it is necessary to deconstruct regional economic development from a more detailed spatial structure and to study the distribution law of sudden environmental accidents in different economic zones employing the eight comprehensive economic zones [28].

The number of sudden environmental accidents accounted for the highest with 33.94% in the eastern coastal economic zone from 2008 to 2022 (Table 4). The middle Yellow River economic zone and the Southwest economic zone were followed by 15.39% and 13.02%, respectively. The Northeast economic region accounted for the smallest proportion, only 2.93%. Because the eastern coastal economic zone is relatively developed economic zone, there are relatively many chemical risk enterprises, high environmental risks, and many branches and tributaries, which is easy for pollution to spread [29]. In the northeast, there are fewer venture enterprises and relatively single traffic conditions, the environmental risk is relatively low.

**Table 4:** Frequency and proportion of sudden environmental accidents occurred in eight economic zones of China from 2008 to 2022.

Area	Times	Proportion/%
Northeast economic zone	164	2.93
Northern coastal economic zone	455	8.15
Eastern coastal economic zone	1895	33.94
Southern coastal economic zone	478	8.56
Middle Yellow River economic zone	859	15.39
Middle Yangtze River economic zone	610	10.93
Southwest economic zone	727	13.02
Northwest economic zone	395	7.08

#### 3.2.2 Inter-provincial characteristics of sudden environmental accidents.

**Fig 2:**
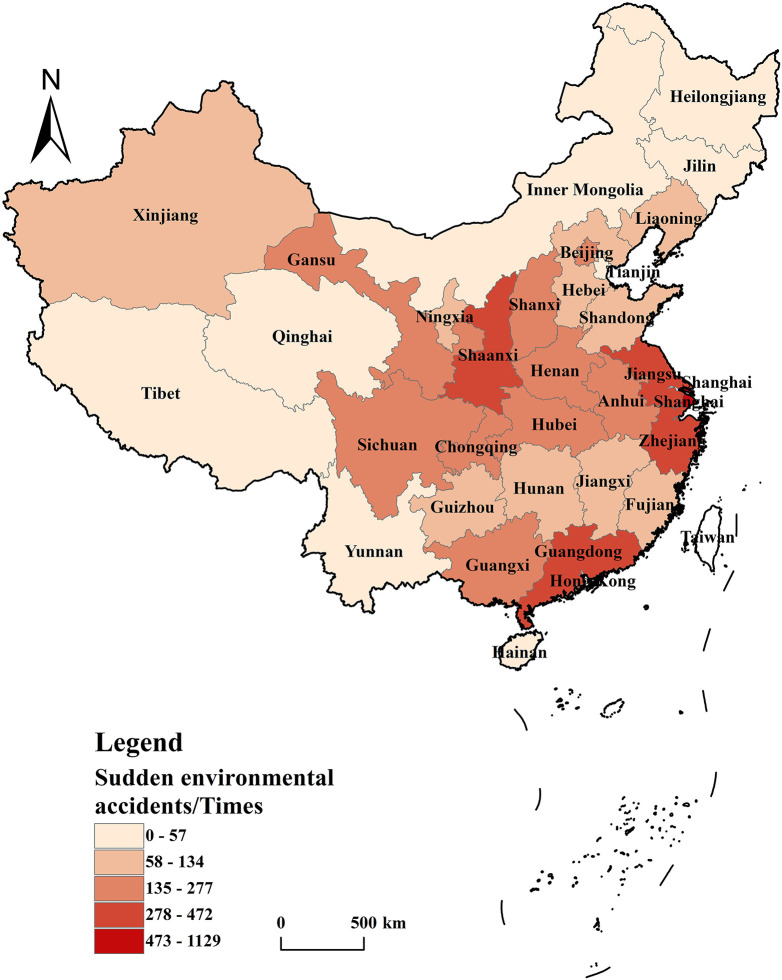
Total of sudden environmental accidents in various provinces of China from 2008 to 2022. GS(2020)4619. The global Moran’s I for sudden environmental accidents was calculated for the period 2008–2022 using Geoda, with the expected value derived under random permutation. The expected value E(I) under randomization was −0.0333. The temporal variation of Moran’s I can be categorized into two distinct stages: The first stage was from 2008 to 2014,Moran’s I exhibited statistical significance at the 0.1 level, with all values exceeding the random-state expected value E(I). This indicated significant positive spatial autocorrelation in sudden environmental accidents.The second stage was from 2015 to 2021, although all Moran’s I values remained above E(I), they failed to pass the significance test, suggesting a random spatial distribution of sudden environmental accidents during this period (Fig 3).

In China, diverse leading industries across regions give rise to distinct environmental risks. Moreover, the pollutant profiles of different areas vary significantly, resulting in a certain degree of correlation in the occurrence of sudden environmental accidents among regions.To comprehensively analyze the environmental risk characteristics of various regions and facilitate the enhancement of local emergency response capabilities, a detailed examination of the geographic distribution of sudden environmental accidents was carried out. GIS-based spatial analysis was employed to investigate sudden environmental accidents in 31 provinces, autonomous regions, and municipalities from 2008 to 2022. These regions were classified into six grades, ranging from high to low, based on the natural break method (Fig 2).

From 2008 to 2022, Shanghai, Shaanxi, and Jiangsu emerged as the top three administrative divisions in China with the highest number of sudden environmental accidents, recording 1,129, 472, and 419 incidents, respectively. These figures accounted for 20.21%, 8.45%, and 7.50% of the national total. Several factors contribute to this trend: Shanghai and Jiangsu, located in the Yangtze River Economic Belt, host a significant concentration of environmentally risky enterprises. Consequently, the likelihood of sudden environmental accidents increases substantially in the event of operational failures or emergencies. Conversely, Shaanxi Province, situated at the hub of China’s transportation network, faces elevated risks from transportation – related accidents compared to other regions, which significantly contributes to its higher accident count.

**Fig 3:**
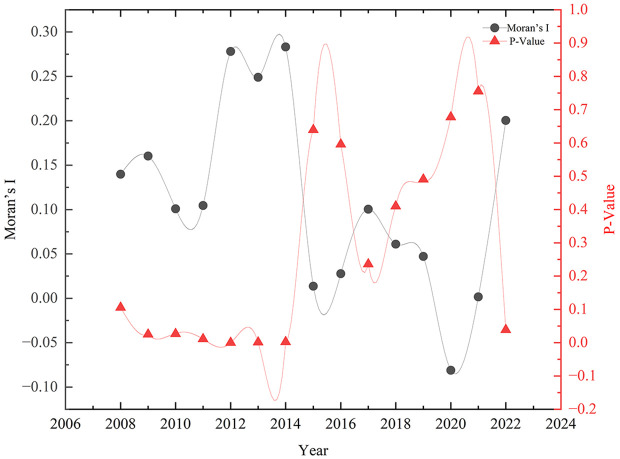
Changes of global Moran index of sudden environmental accidents from 2008 to 2022. The local Moran’s I of provincial sudden environmental accidents could be classified into four types, including Low-Low (L-L), Low-High (L-H), High-Low (H-L), and High-High (H-H), which represents the relationship of sudden environmental accidents between one province and its neighbors. Based on the local Moran’s I of provincial sudden environmental accidents, the local spatial auto correlation of sudden environmental accidents was analyzed from 2008 to 2022. The four clustering types of Moran scatter plots for sudden environmental accidents were visualized for spatial distribution in four years of 2008, 2012, 2017 and 2022 (Fig 4). The local spatial autocorrelation characteristics of sudden environmental accidents are also relatively stable. Provinces which gather High-High (H-H) mainly included Fujian and Jiangxi in 2008, Jiangxi, Hubei, Hunan, Chongqing and Guizhou in 2017, Chongqing, Guizhou and Guangxi in 2022, et al. These provinces with the types of high-high and low-low showed positive spatial correlation. The distribution of sudden environmental accidents is not balanced among provinces. Most provinces with high incidence of sudden environmental accidents will be adjacent to the same nature provinces. The provinces of H-H type are moving from the east to the southwest, the L-L type provinces spread from the east to the central and south, The L-H type provinces are mainly distributed in the western and north-central regions.

**Fig 4:**
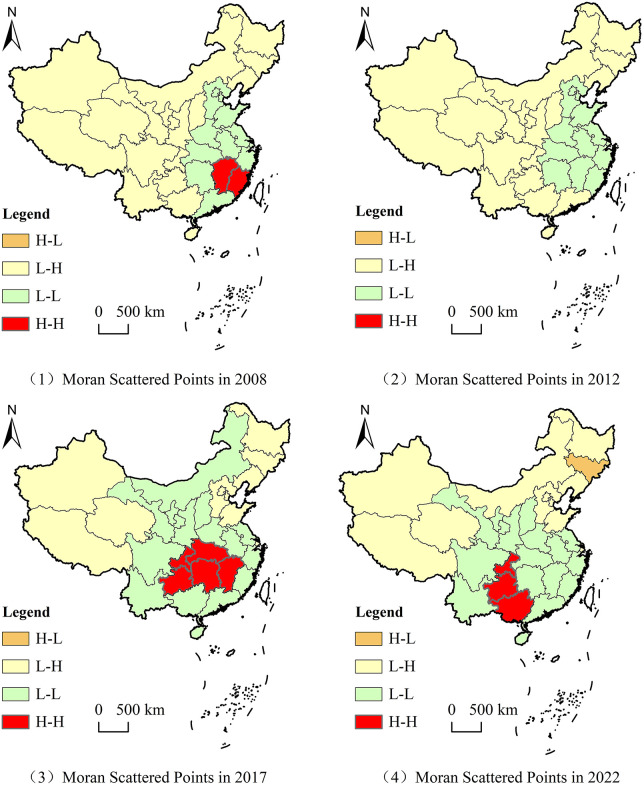
Local spatial autocorrelation aggregation of sudden environmental accidents. GS(2020)4619. All the years have passed the significance test of 1%, sudden environmental accidents showed a positive spatial correlation in most years in the past 15 years. The spatial distribution of sudden environmental accidents is not completely random, and it has obvious spatial dependence in China.

### 3.3 The influencing factors of sudden environmental accidents

**Fig 5:**
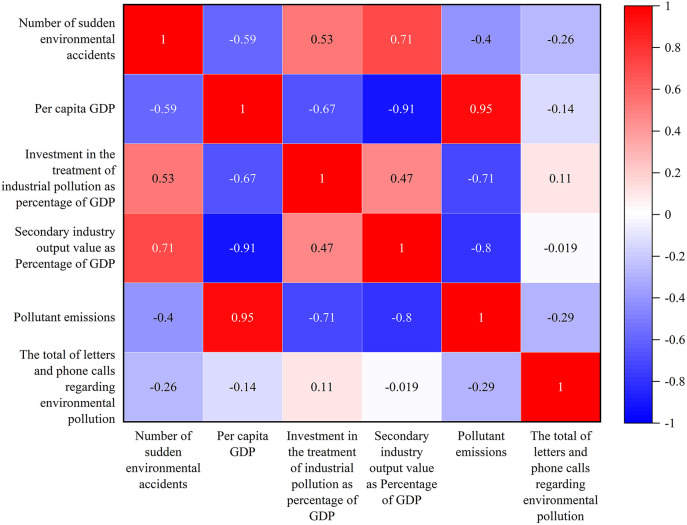
Pearson analysis of related factors of sudden environmental accidents. From 2008 to 2022, the number of sudden environmental accidents exhibited a positive and significant correlation with both industrial pollution treatment investment as a percentage of GDP and secondary industry output value as a percentage of GDP. The investment in industrial pollution control refers to the input allocated to addressing pollution after it has occurred, serving as an ex-post remedial measure. Therefore, a higher ratio of industrial pollution control investment to GDP indicates greater expenditures required for pollution remediation, which in turn reflects a larger number of pollution incidents and a more severe level of pollution. Conversely, it showed a negative and significant correlation with per capita GDP, pollutant emissions, and the total number of environmental pollution-related letters and phone calls.

Sudden environmental accidents, as a subset of environmental emergencies, are influenced by diverse factors. Most production safety accidents are primarily associated with industrial enterprises and economic development, while transportation accidents are linked to cargo turnover, and enterprise pollution is tied to various pollutants in the ecological environment. Therefore, drawing on previous studies [30] and considering data availability, this study selected indicators including per capita GDP, industrial pollution treatment investment as a percentage of GDP, pollutant emissions, secondary industry output value as a percentage of GDP, and the total number of environmental pollution-related letters and phone calls for correlation analysis using Pearson’s correlation coefficient (Fig 5).

## 4. Conclusions

Sudden environmental accidents are characterized by significant causality, rapid spread, and wide incidence. They are likely to cause serious property damage, pose major threats to the economic, social, or political stability of a country or region, and have significant social impacts related to public safety.

(1) The total number of sudden environmental accidents in China exhibited an inverted V-shaped trend, showing an overall downward trajectory. Specifically, it increased from 2008 to 2013 and then declined.
(2) In terms of seasonal distribution, the proportions of sudden environmental accidents from highest to lowest were autumn (29.01%), summer (26.29%), spring (24.80%), and winter (20.19%). The months with the highest frequencies were May and July, while October and December had the lowest. Regarding daily distribution, the 5th (58 cases), 7th (57), 4th (54), 2nd (52), 11th (51), and 9th (50) had the most occurrences, while the 31st (14), 30th (24), 29th (24), and 27th (30) had relatively fewer. By weekday, Monday (16.56%), Wednesday (15.94%), and Thursday (14.38%) had the highest proportions, while Sunday (12.50%) had the lowest.
(3) The spatial distribution of sudden environmental accidents showed an overall imbalance. The highest frequency was observed in the Eastern Coastal Comprehensive Economic Zone, followed by the Middle Reaches of the Yellow River Comprehensive Economic Zone, with the Northeast Comprehensive Economic Zone having the lowest incidence. Provinces with the highest number of sudden environmental accidents were Shanghai (1,129 cases), Shaanxi Province (472 cases), and Jiangsu Province (419 cases). The spatial distribution of sudden environmental accidents in China was not completely random but exhibited spatial correlation. Based on the local Moran’s I of provincial sudden environmental accidents, High-High (H-H) regions were mainly concentrated in the southeastern coastal areas in the early stage, and then gradually shifted westward to the central-southern and even southwestern regions, Low-Low (L-L) areas were clustered in the western and northeastern regions, and Low-High (L-H) areas were distributed in the central regions.
(4) The spatial distribution of sudden environmental accidents was the result of comprehensive influences from multiple factors. Pearson correlation analysis indicated that the main driving factors for sudden environmental accidents in China were industrial pollution treatment investment as a percentage of GDP and secondary industry output value as a percentage of GDP. Per capita GDP, pollutant emissions, and the total number of environmental pollution-related letters and phone calls showed inhibitory effects on the occurrence of sudden environmental accidents, while single factors had relatively minor impacts.

The research analyzed the spatiotemporal evolution characteristics and influencing factors of sudden environmental accidents by employing exploratory spatial analysis and Pearson correlation analysis. The total of sudden environmental accidents has been decreasing in China year by year. However, the frequent and recurring situation of sudden environmental accidents has not thoroughly changed. The spatiotemporal mechanisms of sudden environmental accidents for specific disaster types should be further explored in the future. It is very important to establish more efficient and accurate emergency response management mechanisms. These issues are still to be further analyzed in the future.

## References

[1] The Ministry of Ecology and Environment. Emergency Management Measures of Emergency Environmental Events. 2015.

[2] Liu X, Wang W. The impact of major pollution incidents on environmental performance. Journal of Cleaner Production. 2024;434:139975. doi: 10.1016/j.jclepro.2023.139975

[3] Du L, Wang H, Xu H. Analysis of spatial-temporal association and factors influencing environmental pollution incidents in China. Environmental Impact Assessment Review. 2020;82:106384. doi: 10.1016/j.eiar.2020.106384

[4] Duan J, Mao S, Xie P, Lang J, Li A, Tong J, et al. Key emergency response technologies for abrupt air pollution accidents in China. J Environ Sci (China). 2023;123:235–54. doi: 10.1016/j.jes.2022.03.030 36521987

[5] Bradley MM. NARAC: an emergency response resource for predicting the atmospheric dispersion and assessing the consequences of airborne radionuclides. J Environ Radioact. 2007;96(1–3):116–21. doi: 10.1016/j.jenvrad.2007.01.020 17507121

[6] Zhang JZ, Li SY, Li LL, Zhang M. Thoughts on whole-process environmental risk management in China. Environmental Protection. 2018;46(15):41–3. doi: 10.14026/j.cnki.0253-9705.2018.15.008

[7] Guo QW, Bing YX, Chen SL, Huang DW, Hu LC, Chang S. Pattern of evolution, experience of response, and suggestions of prevention and control of environmental emergencies in China based on typical case studies. Chinese Journal of Environmental Engineering. 2021;15(7):2223–32.

[8] Li QQ, Xie C, Tang HL. Research on emergency monitoring technology for sudden environmental pollution accidents in water bodies. Environment and Development. 2019;31(01):123–5. doi: 10.16647/j.cnki.cn15-1369/X.2019.01.069

[9] Huang L, Huang YJ, Liu PH, Wang G, Bi J. Research on regional comprehensive environmental risk assessment method system. China Environmental Science. 2020;40(12):5468–74. doi: 10.19674/j.cnki.issn1000-6923.2020.0605

[10] Chen ZX, Ding Y, Mao XH, Wang Z, Jia HF. Source identification of accidental water pollution in a tidal river network based on a water environment model and database. Journal of Tsinghua University (Science and Technology). 2017;57(11):1170–8.

[11] Jiang JP, Wang P, Liu J, Yuan YX. Methodological analysis on the research and practices on the warning and emergency response to river chemical spill incidents. Acta Scientiae Circumstantiae. 2017;37(09):3621–8. doi: 10.13671/j.hjkxxb.2017.0089

[12] Li XQ, Mao XQ, Liu SQ. Evaluation methodology of environmental accidents loss and a case study. Ecological Economy. 2011;40(01):24–8.

[13] Cao GZ, Yu F, Wang JN, Zhu WY, Wang D, Zhou XF. Situation, Problems and Countermeasures of Risk Prevention and Control of Environmental Emergencies in the Yangtze River Economic Belt. Chinese Journal of Environmental Management. 2018;10(01):81–5. doi: 10.16868/j.cnki.1674-6252.2018.01.081

[14] Cai ZJ, Liang CY, Zhao SP. Research on emergency environmental accidents grade evaluation. Application Research of Computers. 2014;31(11):3217–20.

[15] Cao G, Yang L, Liu L, Ma Z, Wang J, Bi J. Environmental incidents in China: Lessons from 2006 to 2015. Sci Total Environ. 2018;633:1165–72. doi: 10.1016/j.scitotenv.2018.03.271 29758868

[16] Yu F, Cao GZ, Qi J, Zhao D, Zhang YS, Tian C. Status Quo and prospect of ecological environment risk management and damage compensation in China. Chinese Journal of Environmental Management. 2021;13(05):143–50. doi: 10.16868/j.cnki.1674-6252.2021.05.143

[17] Yu G-H, Wang F-F, Liu X-Z, Li W-H, Xiang Y-B. Spatiotemporal characteristics and influencing factors of environment emergency incident in China from 1991 to 2018. Huan Jing Ke Xue. 2023;44(1):572–82. doi: 10.13227/j.hjkx.202201182 36635845

[18] Li J, Lü Y, He G, Wang T, Luo W, Shi Y. Spatial and temporal changes of emerging environmental pollution accidents and impact factors in China. Huan Jing Ke Xue. 2008;29(9):2684–8. 19068665

[19] Ding L, Huang YL, Liu YL, Liu C, Cheng SG. Spatiotemporal variability of sudden environmental pollution incidents and influencing factors in China, 1995-2012. Progress in Geography. 2015;34(06):749–60.

[20] Li X, Lv JP, Pei YY, Guo CS, Xu J. Analysis of the characteristics of environmental emergencies in China. Journal of Environmental Engineering Technology. 2021;11(02):401–8.

[21] Xu J, Xu M, Zhao Y, Wang S, Tao M, Wang Y. Spatial-temporal distribution and evolutionary characteristics of water environment sudden pollution incidents in China from 2006 to 2018. Sci Total Environ. 2021;801:149677. doi: 10.1016/j.scitotenv.2021.149677 34418617

[22] Yan J, Wang X, Wang T, Zhang X. Research on the spatiotemporal evolution and influencing factors of sudden environmental incidents in China from 2008 to 2021. Pol J Environ Stud. 2024;34(1):891–903. doi: 10.15244/pjoes/186481

[23] Wei B, Liang J, Xie LY, Liu J. Characteristics of environmental emergencies in Gansu Province from 2011 to 2019 and prevention and control countermeasures. Journal of Northwest Normal University (Natural Science). 2021;57(06):124–30.

[24] National Bureau of Statistics of the People’s Republic of China. Chinese Statistical Yearbook. Beijing: China Statistics Press; 2009. 20–22.

[25] National Bureau of Statistics of the People’s Republic of China, Ministry of Ecology and Environment of the People’s Republic of China. China Statistical Yearbook on Environment. Beijing: China Statistics Press; 2009.

[26] Editorial Department of Journal of Safety and Environment. Journal of Safety and Environment. Beijing: Beijing Institute of Technology; 2008. https://navi.cnki.net/knavi/detail?p=Y0gULjk4ovWJ7jKw-F8BpQDpfz-UCmtLLHN6S94Q70NrcEiiWoIjKWz7sCRE0o7icQpxIpNuAjtZYqktwiRe6W_RGlRtbBLkiwRX0Vgplvs=&uniplatform=NZKPT

[27] Yan J, Wang X, Zhang J, Qin Z, Wang T, Tian Q, et al. Research on the spatial and temporal patterns of ozone concentration and population health effects in the Central Plains Urban Agglomeration from 2017 to 2020. PLoS One. 2024;19(5):e0303274. doi: 10.1371/journal.pone.0303274 38753663 PMC11098328

[28] Jie MH, Li LY. Environmental governance performance measurement and spatio temporal evolution of eight comprehensive economic zones in China. Chinese Journal of Population, Resources and Environment. 2023;33(09):86–97.

[29] Sun H, Hu X, Zhao DN, Xie HY, Zhang XJ, Chen C, et al. Status quo and main influencing factors of environmental emergencies in China. Environmental Protection Science. 2022;49(03):130–8. doi: 10.16803/j.cnki.issn.1004-6216.2022070016

[30] Peng LX. Study on the distribution characteristics and influencing factors of sudden environmental emergencies in China during 2011-2020. Modern Chemical Research. 2022;(01):98–101.

